# LINC01089 Inhibits Tumorigenesis and Epithelial–Mesenchymal Transition of Non-small Cell Lung Cancer *via* the miR-27a/SFRP1/Wnt/β-catenin Axis

**DOI:** 10.3389/fonc.2020.532581

**Published:** 2020-11-17

**Authors:** Xingkai Li, Fang Lv, Fang Li, Minjun Du, Yicheng Liang, Shaolong Ju, Zixu Liu, Boxuan Zhou, Bing Wang, Yushun Gao

**Affiliations:** Department of Thoracic Surgery, National Cancer Center/National Clinical Research Center for Cancer/Cancer Hospital, Chinese Academy of Medical Sciences and Peking Union Medical College, Beijing, China

**Keywords:** non-small cell lung cancer, miR-27, SFRP1, EMT, LINC01089

## Abstract

Long noncoding RNAs (lncRNAs) have emerged as regulators of gene expression and play critical regulatory roles in diverse biological functions and diseases, including cancer. In this study, we report the downregulation of LINC01089 in non-small cell lung cancer (NSCLC) samples, relative to adjacent non-tumor tissues, and demonstrate its role in the inhibition of proliferation, migration, and epithelial–mesenchymal transition (EMT) of NSCLC cells. Mechanistic analysis indicates that LINC01089 acts as a sponge for miR-27a, regulating its expression in NSCLC. Interestingly, LINC01089 mediated the upregulation of SFRP1 expression by inhibiting the Wnt/β-catenin–EMT pathway and inhibiting the epithelial–mesenchymal transition of NSCLC *via* sponging miR-27a. Overall, our findings highlight LINC01089’s tumorigenic role and regulatory mechanism in NSCLC, thereby suggesting its potential as a therapeutic target for managing NSCLC.

## Introduction

Lung cancer is one of the most commonly diagnosed cancers in both sexes, accounting for 11.6 and 18.4% of all cancer cases and cancer-related deaths, respectively, worldwide (1). Non-small cell lung cancer (NSCLC) is the most common type of lung cancer, accounting for about 84% of all lung cancer diagnoses. Despite recent diagnostic and therapeutic advances, NSCLC’s 5-year survival rate remains relatively low at less than 18 and 1% for stage IV NSCLC (2). Therefore, to guide the development of more effective therapeutic targets, it is important to elucidate the molecular pathogenesis underlying the initiation and the progression of NSCLC. Rapid advances in genome sequencing have led to the identification of numerous long noncoding RNAs (lncRNAs) (3). Consequently, previous studies have shown that lncRNAs are involved in fundamental biological mechanisms, with a close association to the occurrence of several cancer types, including lung cancer (4), gastric carcinoma (5), prostate cancer (6, 7), and hepatocellular carcinoma (8, 9) among others. Despite their potential as therapeutic targets for cancer treatment, a detailed description of the underlying molecular mechanisms of lncRNAs, especially in NSCLC, remains unclear.

In the present study, we demonstrate the biological function of LINC01089 in non-small cell lung carcinoma, a novel metastasis-inhibiting lncRNA that has been previously associated with poor patient prognosis (10, 11). Our results indicate that LINC01089 was downregulated in NSCLC samples, relative to adjacent non-tumor tissues, and subsequently inhibited the progression of non-small cell lung carcinoma. Functionally, LINC01089 acts as a miR-27a sponge by elevating SFRP1 expression and modulating the Wnt/β-catenin–EMT pathway to arrest non-small cell lung carcinoma tumorigenesis.

## Materials and Methods

### Tissue Sample Collection

A total of 60 pairs of non-small cell lung cancer tumor and adjacent normal tissues were collected from patients at the Department of Thoracic Surgery, Cancer Hospital, Chinese Academy of Medical Sciences and Peking Union Medical College, between November 2018 and January 2019. All samples were obtained before the patients received any form of anticancer treatment. In addition, the patients’ clinicopathologic data were collected and analyzed. The study was approved by the Ethics Committee of the Cancer Hospital, Chinese Academy of Medical Sciences, with surgical samples of primary tumors from lung and lymph nodes staged according to the criteria described by the American Joint Committee on Cancer (8th edition).

### Cell Culture

Human non-small cell lung cancer cell lines and normal epithelial cells were purchased from Shanghai Institute of Biochemistry and Cell Biology (Shanghai, China). These cells were cultured in DMEM or RPMI1640 medium, supplemented with 10% fetal bovine serum, and maintained in a humidified incubator under 37°C and 5% CO_2_ conditions.

### RNA Isolation and Quantitative Real-Time Polymerase Chain Reaction

Total RNA was isolated from the cells using the TRIzol reagent, according to the manufacturer’s instructions. The concentration and the quality of the RNA were measured using a Nanodrop; then, 1 µg was converted to cDNA using the Prime Script RT-PCR kit (Takara, Japan). The cDNA was used as a template for RT-qPCR, targeting the genes below (**Table 1**), with GAPDH also included as an amplification control.

**Table 1:** Primer sequences of the genes used for RT-qPCR.

Gene	Forward 5′-3′	Reverse 5′-3′
**GAPDH**	GGTGAAGGTCGGAGTCAACG	ACCATGTAGTTGAGGTCAATGA
**E-cadherin**	TTCAAAGTGGGCACAGATGGT	TAGGTGGAGTCCCAGGCGTA
**N-cadherin**	AAGGCGTTATGTGTGTATCTTC	TGGAAAGCTTCTCACGGCAT
**Snail**	CTTCGCTGACCGCTCCAACC	GGAGCAGGGACATTCGGGAG
**Slug** **LINC01089** **SFRP1** **miR-27a**	GGCTCATCTGCAGACCCATT AGTCAGGAGCCCTCCTATGG ATGATGATGACAACGACATA TTCACAGTGGCTAAG	TGCTACACAGCAGCCAGATT GGGAGCCAAGGCACTCTAAG ATGCGCTTGAACTCTCTCTGC GTGCAGGGTCCGAGGT
**The sequences of the siRNAs and miRNAs used in transfection**.		
**siRNA**		**Sense 5′-3′**
**siLINC01089-1**		GUCAGGAGCCCUCCUAUGG
**siLINC01089-2**		GAACGUGAGGGUGUAACUU
**siSFRP1**		UGAUGAUGACAACGACAUA
**siNC** **miR-27a mimic** **miR-27a inhibitor** **MiR-NC**		ACGUGACACGUUCGGAGAA UUCACAGUGGCUAAGUUCCGC GCGGAACUUAGCCCACUGUGAA UUGUACUACACAAAAGUACUG
**The sequences of the shRNAs**.		
**shRNA**		**Sense 5'-3'**
**shLINC01089-1**		GTCAGGAGCCCTCCTATGGGC
**shLINC01089-2**		GAACGTGAGGGTGTAACTTAC
**shNC**		ACGTGACACGTTCGGAGAAA

### Cell Transfection

Approximately 2 × 10^5^ of the cells were seeded into six-well plates, based on appropriate cell density. Lipofectamine RNAiMAX was then used for the transient transfection of siRNAs or miRNAs (outlined in **Table 1**), whereas Lipofectamine2000 was employed with plasmid transfection.

### Cell Viability Assay

Approximately 10,000 cells were seeded into a 96-well plate and then transfected with siRNAs, miRNAs, or plasmids, using appropriate protocols. After transfection, CCK-8 reagent was added into each well, followed by incubation and measurement of OD 450 absorbance to determine cell viability.

### Western Blot Assay

Total proteins were extracted from the cells using the RNA immunoprecipitation assay lysis buffer, and then protein concentration was measured using the bicinchoninic acid assay (Pierce). A total of 50 µg of the protein was separated by sodium dodecyl sulfate–polyacrylamide gel electrophoresis, transferred onto nitrocellulose membranes, and then blocked with 5% skim milk. After washing, the membranes were incubated overnight, at 4 °C, with primary β-catenin (ab51032), p-GSK-3*β* (ab93926), GSK-3*β* (ab93926), and GAPDH (ab181602) antibodies. They were washed four times with Tris-buffered saline Tween for 5 min and incubated at room temperature with secondary antibodies for 1 h. GAPDH was used as an internal control.

### Colony Formation Assay

Approximately 1,500 of the treated cells were seeded into six-well plates and cultured for 14 days, with the medium changed after every 3 days. The cells were then fixed using methanol and stained with 0.4% crystal violet solution, and then images were captured using a camera.

### Wound Healing and Transwell Migration Assays

We performed wound healing experiments to determine the cells’ migratory potential. Briefly, a wound was created using a p200 pipette tip on cells grown to confluence using six-well plates; photographs were taken at both 0 and 48 h and then used for determination of cell-free space. Approximately 1 × 10^4^ treated cells were digested into a cell suspension, seeded into 8-µm-pore-size transwells, and then loaded into 24-well plates. The lower and the upper media were supplemented with 10 and 1% FBS, respectively. After 24 h, the transwell was fixed with methanol, the upper cells were scraped using a cotton ball, and the cells at the bottom surface were stained with crystal violet.

### LINC01089 Overexpression, Knockdown, and Virus Production

To overexpress LINC01089, we cloned LINC01089 cDNA into a pLV-puro plasmid (Inovogen Tech. Co., cat. no. VL3001). To produce lentiviruses, HEK-293T cells were transfected with pLV-puro harboring an empty vector or LINC01089 insert, VSVG, and Δ8.9 plasmids for 3 days. The virus was then collected, concentrated by ultracentrifugation (24,000 × *g* for 2 h), and then used to infect cells. After 48 h of infection, the cells were selected by puromycin. To downregulate LINC01089, we cloned shRNA targeting LINC01089 into the pLVshRNA-puro plasmid (Inovogen Tech. Co., cat. no. VL3102). To induce lentivirus production, HEK-293T cells were transfected with pLVshRNA-puro containing shNC or shLINC01089, VSVG, and Δ8.9 plasmids for 3 days. The resulting virus was collected, concentrated by ultracentrifugation (24,000 × *g* for 2 h), and then used to infect cells, with knockdown cells selected using puromycin. The primer sequences of the shRNAs are as shown in **Table 1**.

### Analysis of the Cell Cycle and Apoptosis

Approximately 2 × 10^5^ A549 cells, transfected with siLINC01089, were seeded in six-well plates, for 48 h, and then stained with propidium iodide (PI; Beyotime, Shanghai, China) to assess the cell cycle. Generally, the red area on the left side of the cell cycle plot represents G1 phase cells, whereas the intermediate white and red areas denote the S and G2 phase cells, respectively. Cell apoptosis was detected using annexin V–fluorescein isothiocyanate (FITC) and PI, as described by the manufacturer’s instructions of the FITC Annexin V Apoptosis Detection kit. The harvested cells were further analyzed by flow cytometry (FACScan; BD Biosciences, San Jose, CA, USA) according to the manufacturer’s instructions.

### Dual-Luciferase Reporter Assay

Wild-type or mutated LINC01089 or SFRP1 sequences were synthesized and cloned into the pmirGLO dual-luciferase vector. The cells were seeded into 24-well plates, co-transfected with respective plasmids (LINC01089-WT, LINC01089-mut, SFRP1-WT, and SFRP1-mut) and miR-27a, and then incubated for 48 h. Luciferase activity was thereafter detected using the dual-luciferase reporter system (Promega, E1910).

### RNA Immunoprecipitation Assay

RNA immunoprecipitation (RIP) was performed as previously described (12). To summarize, A549 cells were transfected with miR-NC or miR-27a and then digested using trypsin. The cells were lysed in a lysis buffer [50 mM Tris, pH 7.4, 150 mM NaCl, 0.5% NP-40, 0.5 mM phenylmethylsulfonyl fluoride (PMSF), 2 mM ribonucleoside vanadyl complex (RVC), protease inhibitor cocktail (Roche)], the supernatant was collected, and then approximately 10% of the cell extract was taken out as input. The remaining part was co-precipitated with rabbit anti-AGO2 (ab186733, 5 µg) or IgG (ab109489, 5 µg) antibodies. The magnetic beads–antibody complex was washed four times with RIP wash buffer [50 mM Tris, pH 7.4, 300 mM NaCl, 0.05% sodium deoxycholate, 0.5% NP-40, 0.5 mM PMSF, 2 mM RVC, protease inhibitor cocktail (Roche)] and then eluted with 100 µl of cell elution buffer. After detachment using proteinase K, RNA was isolated from the samples as well as input for subsequent PCR quantification.

### RNA Pull‐Down Assay

The biotin-labeled RNA (miR-27a probe sequence: TTCACAGTGGCTAAGTTCCG) was in combination with streptavidin C1 Dynabeads (cat: 65002; Invitrogen) according to the manufacturer’s instructions. The cells were harvested and lysed with lysis buffer (Ambion, Austin, TX). The lysate was incubated with bio-RNA–beads complex overnight at 4°C on a rotating platform. After washing with wash buffer four times, the bound RNA was extracted with Trizol, and LINC01089 enrichment was determined by RT-qPCR.

### Bioinformatics Analyses

Patterns of LINC01089 expression, between NSCLC and normal tissues, as well as the overall survival of patients were analyzed using the Gene Expression Profiling Interactive Analysis (GEPIA) database (http://gepia.cancer-pku.cn/). Generally, GEPIA is a web-based tool that delivers fast and customizable functionalities based on The Cancer Genome Atlas and Genotype-Tissue Expression databases. Functionally, it provides key interactive and customizable functions, including differential expression, profile plotting, and patient survival analyses among others (13). Based on the resultant profiles of LINC01089 expression, we selected the top and the bottom 50% as the high and the low expression groups, respectively. Thereafter, Kaplan–Meier survival curves were plotted for each of the patient groups, and statistical significance of the prognosis difference was inferred using log-rank test.

### *In Vivo* Animal Experiments

A total of 12 BALB/c athymic nude mice were divided into two cohorts (*n* = 6 per group). Approximately 4 × 10^6^ of PC9 cells that were transduced with pLV-LINC01089 or pLV-Empty were collected, suspended in 100 μl Hank’s balanced salt solution (HBSS) buffer, and then subcutaneously injected into the mice’s right flank. A separate cohort, comprising 18 BALB/c athymic nude mice, was divided into three groups (*n* = 6 per group). Approximately 1 × 10^6^ A549 cells, earlier stably transduced with shRNA targeting LINC01089 or shNC, were collected, suspended in 100 μl HBSS buffer, and then subcutaneously injected into the mice’s right flank. Tumor volumes were measured every 7 days and calculated as length × width × width × 0.5. After 4 weeks, the mice were sacrificed, and the tumors were weighed. To determine metastasis, luciferase-labeled A549 cells were first stably transduced with shRNA targeting LINC01089 or shNC, and then 3 × 10^6^ of the cells were injected into the tail vein of BALB/c athymic nude mice. IVIS-200 imaging system was then used to regularly monitor metastatic growth *in vivo*. At completion of the experiments, the animals were sacrificed, and their lungs were collected for further analysis.

### Statistical Analyses

All statistical analyses were performed using Graphpad Prism software. Data, for at least three independent experiments, were expressed as means ± standard deviations (SD) of the mean. Pearson correlations were used to determine the relationship between miR-27a and LINC01089 or SFRP1, whereas Student’s *t*-test was used to compare differences between experimental and control groups. Data followed by *P* < 0.05 were considered statistically significant.

## Results

### LINC01089 Was Downregulated in NSCLC Tissues and Cells

**Figure 1:**
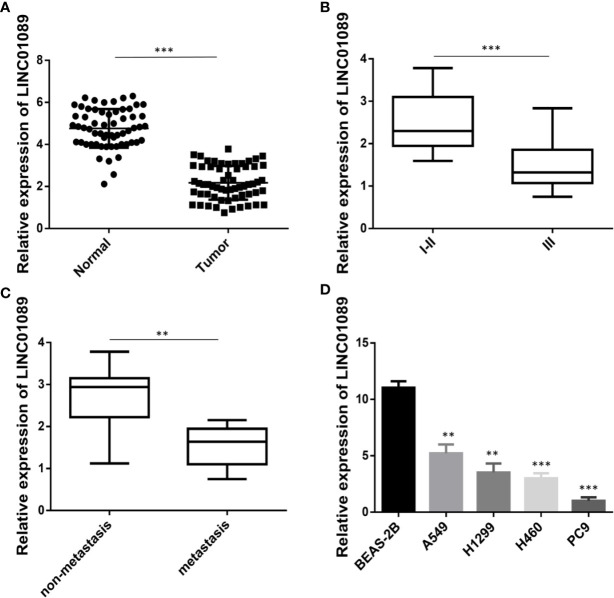
LINC01089 was down-expression in NSCLC tissues. **(A)** LINC01089 expression was detected by RT-qPCR in NSCLC tissues and para-carcinoma tissues. **(B)** The expression level of LINC01089 between stage (I–II) and stage (III) in NSCLC tissues. **(C)** RT-qPCR analysis of relative mRNA expression of LINC01089 in lymph nodes non-metastasis and metastasis samples of NSCLC. **(D)** LINC01089 expression was detected in NSCLC cell lines (A549, H1299, H460, PC9) and human normal lung epithelial cell line (BEAS-2B). Data are reported as means ± SD. **P < 0.01; ***P < 0.001.

To determine levels of LINC01089 expression in non-small cell lung cancer, qRT-PCR was performed on clinical specimens. The results showed significantly lower levels in NSCLC than adjacent normal tissues (**Figure 1A**). Moreover, a search on the GEPIA database revealed a downregulation of LINC01089 expression in both lung adenocarcinoma and lung squamous carcinoma samples, relative to the adjacent normal tissues (**Figure S1A**, **Figure S1B**). The 60 NSCLC patients enrolled in our study revealed two (relatively high and low expression) groups based on the median level of LINC01089 expression in NSCLC tissues. Moreover, an analysis of the relationship between LINC01089 expression and the clinicopathological features in the NSCLC samples showed that low LINC01089 levels were significantly correlated with TNM classification (*p* = 0.035), lymph node metastasis (*p* = 0.0307), and poor differentiation (*p* = 0.0044) (**Table 2**, **Figures 1A–C**). Furthermore, Kaplan–Meier curves and log-rank test, based on the GEPIA database, revealed that NSCLC patients with lower LINC01089 expression had poor overall survival (hazard ratio = 0.68, *P* = 0.0067, **Figure S1C**). Besides that, LINC01089 expression was downregulated in NSCLC cell lines (A549, H1299, H460, and PC9) compared with human normal lung epithelial cell lines (BEAS-2B) (**Figure 1D**). These results indicated that LINC01089 was involved in the development and the progression of NSCLC and could, therefore, serve as a potential prognosis biomarker in NSCLC patients.

**Table 2:** Correlation between Linc01089 expression and clinicopathological features in NSCLC patients (n=60).

Characteristics	Number	Linc01089		*P-*Value
		High expression (n = 18)	Low expression (n = 42)	
Age (year)	60	59.83±7.97	58.45±8.61	0.5630
Gender (n, %)				0.5501
Female	40	11 (27.50)	29 (72.50)	
Male	20	7 (35.00)	13 (65.00)	
Smoking				0.6492
Y	26	7 (26.92)	19 (73.08)	
N	34	11 (32.35)	23 (67.65)	
TNM stage (n, %)				0.0355
I-II	44	17 (38.64)	27 (61.36)	
III	16	1 (6.25)	15 (93.75)	
Lymph node metastasis (n, %)				0.0307
Y	26	4 (15.38)	22 (84.62)	
N	34	14 (41.18)	20 (58.82)	
Differentiation (n, %)				0.0044
Well moderate	41	17 (41.46)	24 (58.54)	
Poor	19	1 (5.26)	18 (94.74)	
Histologic Type				0.4519
Adenocarcinoma	47	13 (27.66)	34 (72.34)	
Squamous carcinoma	13	5 (38.46)	8 (61.54)	

### LINC01089 Suppressed the Migration of NSCLC Cells *In Vitro* and *In Vivo*

**Figure 2:**
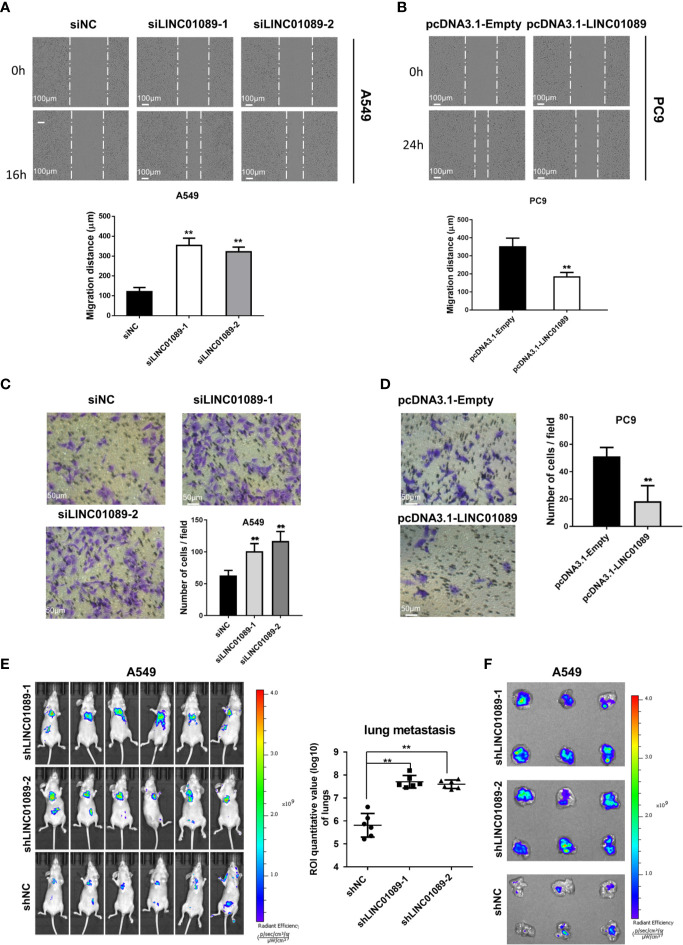
LINC01089 suppressed the migration of NSCLC cells. **(A)** Effects of siLINC01089 on A549 cells migration by wound-healing assay. Data are quantification below. **(B)** Effects of pcDNA3.1-LINC01089 on PC9 cells migration by wound-healing assay. Data are quantification below. **(C)** Cell migration capacity was validated in siLINC01089-transfected A549 cells. **(D)** Cell migration ability was detected in in pcDNA3.1-LINC01089-transfected PC9 cells. **(E)** Luciferase signal intensities of the mice in each group were shown, and ROI area (lung) was measured and analyzed statistically. **(F)** Representative lungs from **(E)**. Data are reported as means ± SD. **P < 0.01.

To determine LINC01089’s biological functions in NSCLC cells, we transfected A549 and H1299 cells with LINC01089 small interfering RNA (siRNA) targeting LINC01089 or LINC01089 plasmid (pcDNA3.1-LINC01089), respectively, and then analyzed the efficiency of knockdown and overexpression of LINC01089 using qRT-PCR at 48 h after transfection (**Figure S2A**, **Figure S2B**). The wound healing assay revealed a significantly higher cell migration ability in siLINC01089-transfected A549 than siNC-transfected A549 cells (**Figure 2A**). Similarly, pcDNA3.1-LINC01089-transfected PC9 cells exhibited a significantly lower migration ability than those transfected with pcDNA3.1-Empty (**Figure 2B**). To further examine the effect of LINC01089 on cell migration, siLINC01089-transfected A549 and pcDNA3.1-LINC01089-transfected PC9 cells were cultured on a transwell membrane apparatus. The results from the transwell assays showed that LINC01089 knockdown significantly increased the migratory abilities of A549 cells (**Figure 2C**), whereas its overexpression dramatically inhibited the migratory activities of PC9 cells (**Figure 2D**). We also knocked down LINC01089 in luciferase-expressing A549 cells with shRNA and then intravenously injected the cells into nude mice to establish tumor metastasis. *In vivo* imaging data showed that LINC01089 knockdown induced more metastasis in the lungs (**Figures 2E, F**). Taken together, these results indicated that LINC01089 could inhibit NSCLC metastasis, both *in vitro* and *in vivo*.

### LINC01089 Suppresses the Proliferation of NSCLC Cells, Both *In Vitro* and *In Vivo*

**Figure 3:**
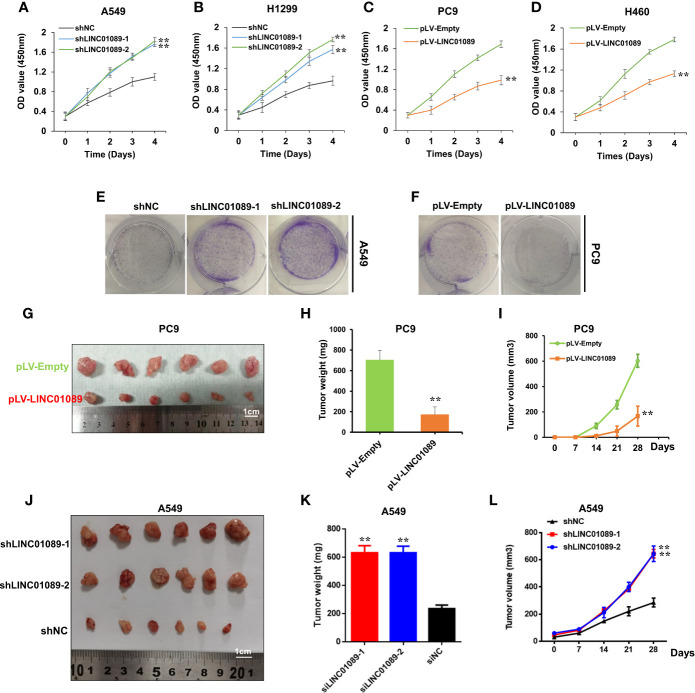
LINC01089 suppressed the proliferation of NSCLC cells *in vitro* and *in vivo*. **(A)** Cell proliferation viability was validated in shLINC01089-transfected A549 cells. **(B)** Cell proliferation viability was validated in shLINC01089-transfected H1299 cells. **(C)** Cell proliferation viability was validated in pLV-LINC01089-transfected PC9 cells. **(D)** Cell proliferation viability was validated in pLV-LINC01089-transfected H460 cells. **(E)** Clonogenicity assay of the effect of LINC01089 after transfection with shLINC01089 in A549 cells. **(F)** Clonogenicity assay of the effect of pLV-LINC01089 after transfection with pLV-LINC01089 in PC9 cells. **(G)** The size of xenograft tumors in the pLV-LINC01089 group (down) and the pLV-Empty group (up) (cm). **(H)** The weight of the xenograft tumors in the pLV-LINC01089 group and the pLV-Empty group. **(I)** The growth curve of the xenograft tumors in the pLV-LINC01089 group and the pLV-Empty group. **(J–L)** The size, weight, and growth curve of xenograft tumors in the sh-LINC01089 group and the shNC group were measured and analyzed. Data are reported as means ± SD. **P < 0.01.

To further elucidate LINC01089’s role in the proliferation of NSCLC cells, we stably expressed LINC01089 by infecting the cells using a lentivirus. Specifically, A549 and H1299 cells were transduced with shRNA, targeting LINC01089, to knock down its expression (**Figure S3C**), whereas PC9 and H460 cells were transduced with pLV-LINC01089 plasmid to overexpress it (**Figure S3E**). Results from the CCK-8 assay revealed high proliferation in siLINC01089-transfected A549 cells (**Figure S3A**) as well as A549 and H1299 cell lines (**Figures 3A, B****)**. However, lower viability was recorded in pcDNA3.1-LINC01089-transfected PC9 cells (**Figure S3B**) as well PC9 and H460 cell lines (**Figures 3C, D**). On the other hand, knocking down LINC01089 did not affect the growth of normal cells (BEAS-2B) (**Figure S3C**, **Figure S3D**). Moreover, the clonogenic assay showed that LINC01089 knockdown significantly increased the colony-forming efficiency of A549 cells (**Figure 3E**, **Figure S2C**), while overexpressing LINC01089 together with pLV-LINC01089 transduction significantly reduced the colony-forming efficiency of PC9 cells (**Figure 3F**, **Figure S2D**). To further examine the role of LINC01089 on the proliferation of NSCLC cells *in vivo*, we transduced PC9 cells with pLV-LINC01089 or pLV-Empty. The results showed a stable overexpression of LINC01089 in pLV-LINC01089-transduced PC9 cells (**Figure S3E**). Subcutaneous injection of the transduced cells into nude mice resulted in significantly lower growth of tumors in xenografts in the pLV-LINC01089 group relative to those in the pLV-Empty (**Figures 3G–I**), an indication that LINC01089 could suppress tumor growth *in vivo*. Thereafter, subcutaneous injection of shLINC01089-A549 into nude mice, for establishment of tumor proliferation, showed that depletion of LINC01089 promoted tumorigenesis in *vivo* (**Figures 3J–L**).

### LINC01089 Promotes Cell Cycle Progression

**Figure 4:**
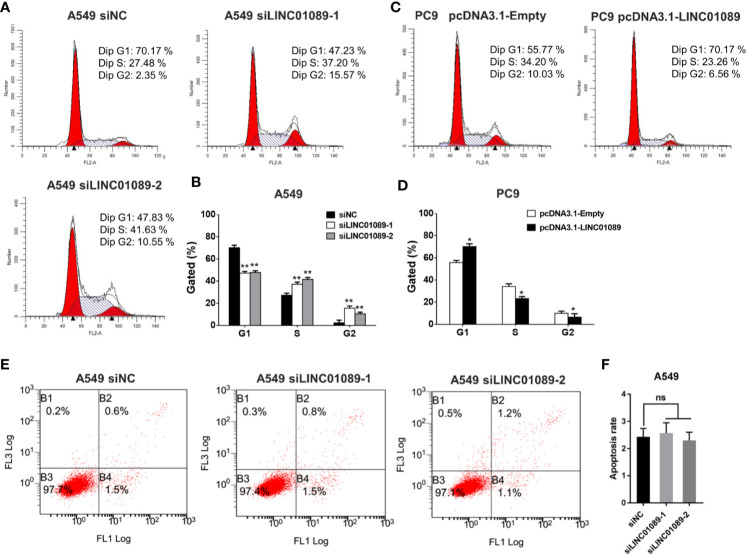
LINC01089 promoted NSCLC cell cycle progression. **(A)** Flow cytometry was performed to determine the effects of siLINC01089 on cell cycle in A549 cells. In the cell cycle plot, the red area on the left represents G1-phase cells, the intermediate white area represents S phase cells, and the red area on the right represents G2 phase cells. **(B)** Quantification of A. **(C)** Flow cytometry was performed to determine the effects of pcDNA3.1-LINC01089 on cell cycle in PC9 cells. **(D)** Quantification of C. **(E)** Apoptosis was analyzed by flow cytometry in siLINC01089-transfected A549 cells **(F)** Quantification of E. Data are reported as means ± SD. *P < 0.05; **P < 0.01.

Flow cytometry was used to determine the effect of knocking down or overexpressing LINC01089 on cell cycle or apoptosis in NSCLC cells. The results indicated that downregulating LINC01089 promoted the transition from G1 phase to S phase of the cell cycle (**Figures 4A, B**). Conversely, its overexpression reduced the number of cells in the S phase and generated an unstable number of cells in the G2 phase (**Figures 4C, D**). An analysis of LINC01089’s effect on cell apoptosis, using annexin V–FITC assays, revealed no significant differences in the proportion of apoptotic cells between the experimental and the control groups after a 48-h period (**Figures 4E, F**). Overall, these results indicated that the LINC01089-mediated promotion of NSCLC cell proliferation was due to cell cycle modulation and not apoptosis.

### LINC01089 Functions as a ceRNA for miR-27a in NSCLC

**Figure 5:**
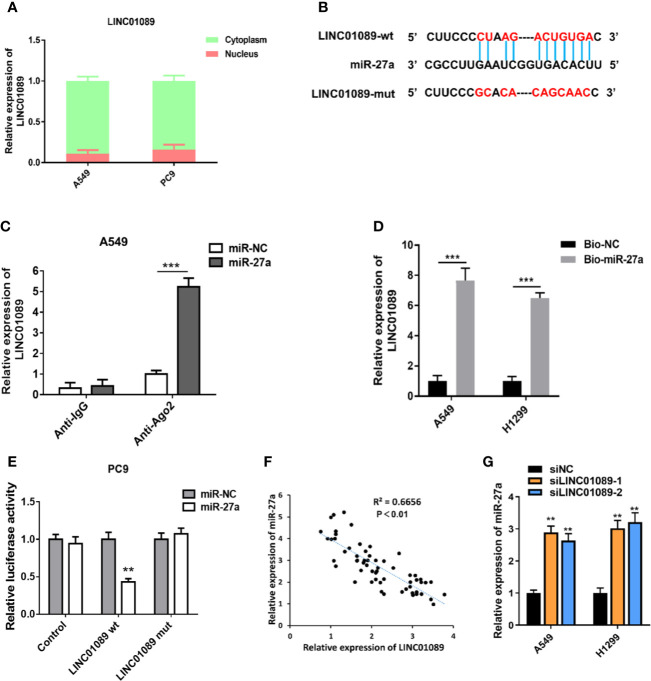
LINC01089 functioned as a ceRNA for miR-27a in NSCLC. **(A)** Subcellular localization of LINC01089 was detected through nucleocytoplasmic separation assay. **(B)** A schematic outline of sequence sites of miR-27a targeted to LINC01089 predicted by bioinformatic analysis. **(C)** The association between LINC01089 and miR-27a was evaluated by Ago2-RIP assay. RNA immunoprecipitation (RIP) experiments were performed in A549 cells overexpressing miR-27a or miR-NC. **(D)** RNA pull-down assay was used to detect the direct interaction between miR-27a and LINC01089. **(E)** Dual-luciferase reporter assay was carried out to examine the luciferase activity of LINC01089-wt group and LINC01089-mut group. **(F)** The relationship between LINC01089 expression and miR-27a expression was examined by Spearman correlation analysis in 60 NSCLC tissues. **(G)** The expression of miR-27a was detected in siLINC01089 transfected A549 and H1299 cells. Data are reported as means ± SD. **P < 0.01; ***P < 0.001.

To elucidate LINC01089’s regulatory mechanism in NSCLC, we analyzed its subcellular localization in NSCLC cells, with results from the nucleocytoplasmic separation assay showing that LINC01089 was mainly localized in the cytoplasm (**Figure 5A**). Prediction of LINC01089’s potential targets, using StarBase V3.0, revealed that the lncRNA contains complementary sequences that bind miR-27a (**Figure 5B**). To further verify the direct binding between miR-27a and LINC01089 at endogenous levels, we performed RIP experiments in the A549 cell line that had earlier been transfected with miR-NC or miR-27a using the Ago2 protein, a key component of the microRNA-containing RNA-induced silencing complex. The results revealed a significant enrichment of LINC01089 immunoprecipitated by anti-Ago2 antibody in miR-27a overexpression cells (**Figure 5C**). Additionally, results from a biotin-labeled system, used to determine whether miR-27a could pull down LINC01089, revealed significantly high levels of LINC01089 in the pull-down product isolated from A549 and H1299 cells, following transfection with biotin-labeled miR-27a, relative to the control group (**Figure 5D**). This suggested that miR-27a interacted with LINC01089 in a sequence-specific manner. To further determine whether LINC01089 directly bound miR-27a, we performed a dual-luciferase reporter assay by co-transfecting LINC01089-wt or LINC01089-mut containing target sequences and miR-27a in PC9 cells. We found a significantly lower luciferase activity in LINC01089-wt relative to the LINC01089-mut group (**Figure 5E**). Moreover, Spearman’s correlation analysis revealed an inverse relationship between LINC01089 and miR-27a expression (**Figure 5F**). Furthermore, qRT-PCR analysis indicated that knocking down LINC01089 in A549 and H1299 cells mediated miR-27a mRNA expression (**Figure 5G**).

### miR-27a Promoted EMT of NSCLC *via* the SFRP1-Wnt/β-catenin Pathway

**Figure 6:**
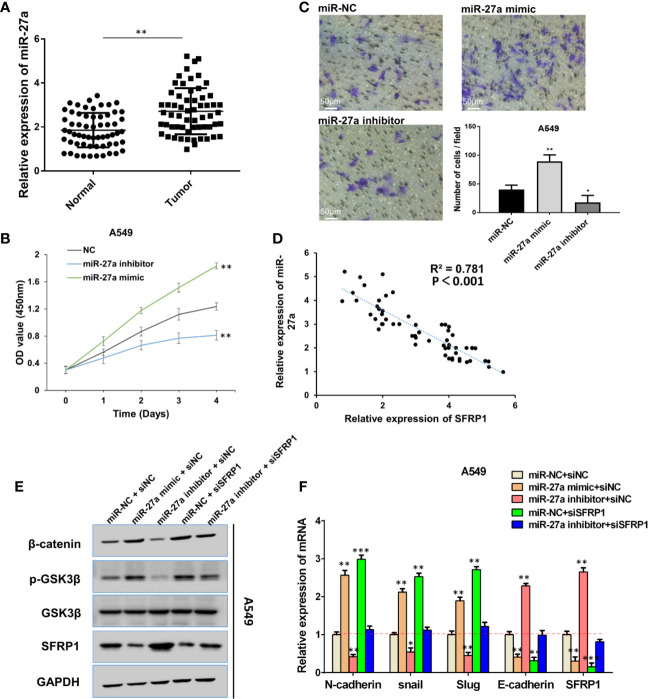
MiR-27a promoted EMT progression of NSCLC via SFRP1- Wnt/β-catenin pathway. **(A)** RT-qPCR was performed to detect miR-27a expression in NSCLC tissues and para-carcinoma tissues. **(B)** The function of miR-27a on cell proliferation viability was examined by CCK-8 assay. **(C)** Migration capacity was performed by transwell assay in miR-27a mimics/inhibitor-transfected A549 cells. **(D)** Spearman correlation analysis of the correlation between SFRP1 and miR-27a expression in 60 NSCLC tissues. **(E)** Western blot assay detected the protein level of β-catenin, SFRP1, p-GSK3β and GSK3β, GAPDH acting as reference gene. **(F)** mRNA levels of EMT markers were detected in A549 cells by RT-qPCR. Data are reported as means ± SD. *P < 0.05; **P < 0.01; ***P < 0.001.

Despite previous studies demonstrating microRNA-27a’s role as an oncogenic miRNA in multiple tumor progression (14, 15), its function in lung cancer remains unclear. In the present study, qRT-PCR revealed significantly higher miR-27a levels in NSCLC relative to adjacent normal tissues (**Figure 6A**). Moreover, A549 cells were transfected with miR-27a inhibitor or mimics, and transfection efficiency was assessed by qRT-PCR (**Figure S4**). The CCK-8 assay revealed significantly higher and lower proliferation in miR-27a mimic-transfected and inhibitor-transfected A549 cells, respectively (**Figure 6B**). The transwell assay showed that miR-27a overexpression significantly enhanced cell migration ability, whereas its knockdown significantly inhibited this phenomenon (**Figure 6C**). Taken together, these results suggested that miR-27a promotes NSCLC cell viability and migration.

Previous studies have shown that miR-27a modulates gastric cancer cell metastasis by promoting the epithelial–mesenchymal transition (EMT) pathway in gastric cancer (16) and targets SFRP1 to regulate the proliferation and the migration of osteosarcoma cells (17). In fact, SFRP1 suppressed tumor progression through epithelial-to-mesenchymal transition *via* the Wnt/β-catenin signaling pathway (18). In the present study, Spearman’s correlation analysis showed that miR-27a expression was inversely associated with SFRP1 expression in NSCLC tissue samples (**Figure 6D**), corroborating the findings that SFRP1 is negatively regulated by miR-27a. To ascertain whether SFRP1 is involved in miR-27a-related EMT, *via* the Wnt/*β*-catenin pathway in NSCLC cells, we co-transfected mimics and inhibitors of miR-27a with siSFRP1 in A549 and H1299 cells. The results indicated that mimic-transfected A549 cells increased the expression of p-GSK-3P and β-catenin, whereas miR-27a inhibitors showed a decreased expression of p-GSK-3P and β-catenin (**Figure 6E**). In addition, silencing of SFRP1 enhanced p-GSK-3P expression, while β-catenin was impaired by miR-27a inhibitor (**Figure 6E**). Moreover, detection of EMT markers through qRT-PCR revealed significantly higher levels of Slug, Snail, and N-cadherin and decreased SFRP1 and E-cadherin expression in cells expressing miR-27a mimic (**Figure 6F**). Similarly, silencing SFRP1 blocked the inhibitory effect of miR-27a inhibitor on Slug, Snail, and N-cadherin and downregulated E-cadherin. Overall, these results indicated that miR-27a promoted EMT in NSCLC *via* the SFRP1-Wnt/β-catenin signaling pathway.

### LINC01089 Suppresses EMT in NSCLC *via* the miR-27a–SFRP1-Wnt/β-Catenin Axis

**Figure 7:**
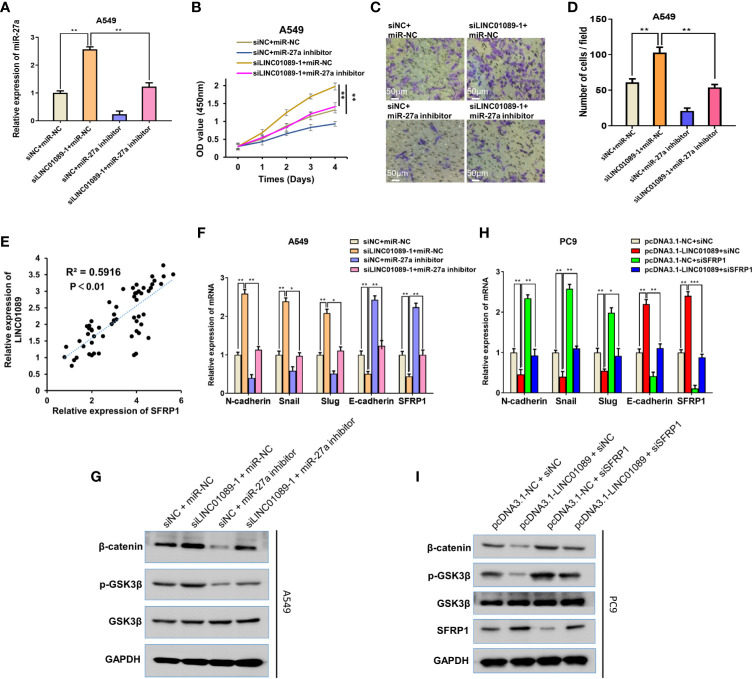
LINC01089 suppresses EMT of NSCLC via miR-27a-SFRP1-Wnt/β-catenin axis. **(A)** miR-27a expression was validated in A549 cells transfected with inhibitor and/or siLINC01089 by RT-qPCR **(B, C)** A549 cells were transfected with miR-27a mimics and/or siLINC01089, CCK-8 and transwell assays were to detect the proliferation viability and migration capability. **(D)** Quantification of C **(E)** Spearman correlation was used to analyze the correlation between LINC01089 and SFRP1 expression in 60 NSCLC tissues. **(F)** RT-qPCR was used to analyze the mRNA levels of N-cadherin, Snail, Slug and SRPR1 in A549 cells cotransfected with miR-27a inhibitor and/or siLINC01089. **(G)** Western blot was used to analyze the protein levels of p-GSK-3P and β-catenin in A549 cells cotransfected with miR-27a inhibitor and/or siLINC01089. GAPDH acted as reference gene. **(H)** RT-qPCR was used to analyze the mRNA levels of N-cadherin, E-cadherin, Snail, Slug, and SRPR1 in PC9 cells cotransfected with pcDNA3.1-LINC01089 and/or siSFRP1. **(I)** Western blot was used to analyze the protein levels of p-GSK-3P and β-catenin in PC9 cells cotransfected with pcDNA3.1-LINC01089 and/or siSFRP1. GAPDH acted as reference gene. Data are reported as means ± SD. *P < 0.05; **P < 0.01; ***P < 0.001.

To further explore the mechanism underlying LINC01089, we assessed whether LINC01089 could suppress EMT in NSCLC *via* targeting the miR-27a–SFRP1-Wnt/β-catenin axis. Specifically, we performed rescue experiments to verify the hypothesis and found that siLINC01089 or siNC was successfully co-transfected with miR-NC or miR-27a inhibitor in NSCLC cells (**Figure 7A**). The miR-27a inhibitor successfully reversed the LINC01089 knockdown-mediated increase in miR-27a expression. After LINC01089 knockdown, the miR-27a inhibitor reduced cell proliferation and migration (**Figures 7B, D**), indicating that the oncogenic function of LINC01089 might be dependent on miR-27a. Additionally, the mRNA expression of LINC01089 was positively correlated with SFRP1 (**Figure 7E**).

LINC01089 mediated a reduction in EMT progression of NSCLC cells *via* the Wnt/β-catenin signaling pathway. To summarize, LINC01089 knockdown mediated an increase in mRNA level of N-cadherin, E-cadherin, Snail, Slug, and SRPR1 (**Figure 7F**), with protein levels of p-GSK-3P and β-catenin also found to increase following transfection with siLINC01089 in A549 and H1299 cells (**Figure 7G**, **Figure S5**). Interestingly, miR-27a inhibitor reversed the effect of LINC01089 knockdown and elevated the expression of N-cadherin, E-cadherin, Snail, Slug, and SRPR1 at mRNA levels (**Figure 7F**). Additionally, miR-27a inhibitor reversed the effect of LINC01089 knockdown and mediated an increase in p-GSK-3P and β-catenin expression at the protein level (**Figure 7G**, **Figure S5**). Overexpressing LINC01089 resulted in low mRNA levels of N-cadherin, Snail, Slug, and SRPR1 (**Figure 7H**), whereas the protein expression of p-GSK-3P and β-catenin also reduced following transfection with pcDNA3.1-LINC01089 in A549 cells (**Figure 7I**). Moreover, SFRP1 knockdown rescued the effect of LINC01089 overexpression, which inhibited the expression of N-cadherin, Snail, Slug, and SRPR1 at the mRNA levels (**Figure 7H**), while its knockdown rescued the effect of LINC01089 overexpression and inhibited the expression of p-GSK-3P and β-catenin at the protein level (**Figure 7I**). Taken together, these results demonstrate that LINC01089 suppressed NSCLC tumorigenesis and progression by inhibiting the miR-27a–SFRP1-Wnt/β-catenin–EMT pathway.

## Discussion

Despite the recent advancements in cancer treatment over the last decade, no effective treatment therapies for NSCLC patients are currently available (19), necessitating the identification of novel potential target genes to guide the elucidation of the underlying mechanisms of NSCLC development. Accumulating evidence shows that lncRNAs play a critical role in tumorigenesis of multiple cancers, including NSCLC, although only a handful of lncRNAs have been characterized with regards to their function. In the present study, we explored the roles of LINC01089 in lung carcinoma progression and unraveled the associated underlying mechanisms. LINC01089 is a highly conserved lncRNA that inhibits cell proliferation and causes metastasis in breast cancer. However, its function in other cancer types, especially non-small cell lung cancer, remains unclear. The results of the present study revealed that LINC01089 was significantly downregulated in NSCLC tissues and cell lines, relative to adjacent normal tissues and cells. Subsequently, an analysis of LINC01089 on cell proliferation and migration in NSCLC *in vitro* and in mouse tumor formation revealed that LINC01089 knockdown promoted the cell proliferation and the migration as well as tumor formation in NSCLC. Taken together, these results indicated that LINC01089 acts as a metastasis-inhibiting marker during NSCLC progression, consistent with the function previously reported in breast cancer.

One of the major mechanisms for lncRNAs is that they function as competing endogenous RNA, where they bind miRNAs and generate a posttranscriptional function in mRNA targets associated with tumorigenesis (20–23). In the present study, we determined whether LINC01089 functions as a competing endogenous RNA in NSCLC by analyzing its subcellular localization in cell line. Our results indicated that LINC01089 was mainly localized in the cytoplasm and had a direct interaction with miR-27a. Previous studies have reported the oncogenic role played by miR-27a in prostate (24), thyroid (25), gastric (26), and breast (27) cancers. The results of the present study further indicated that SFRP1 was a direct target of miR-27a in NSCLC. Previous studies have shown that SFRP1 is an antagonist of the Wnt signaling pathway, where it interrupts the interaction between Wnt glycoprotein (ligand) and frizzled receptor, thereby preventing tumorigenesis (28, 29). Since EMT is well known as a classic cell phenotype that is frequently involved in the regulation of cancer cell progression, we hypothesized that LINC01089 suppressed non-small cell lung carcinoma EMT progression by sponging miR-27a to target the SFRP1-Wnt/β-catenin pathway. Specifically, LINC01089 suppressed NSCLC tumorigenesis by regulating the miR-27a–SFRP1-Wnt/β-catenin–EMT pathway.

## Conclusion

In summary, our results indicate that LINC01089 is a tumor suppressor in NSCLC. Functionally, LINC01089 plays an essential role in cell proliferation and migration by functioning as a ceRNA of miR-27a and subsequently inhibiting the SFRP1-Wnt/β-catenin signaling pathway. This affirms its potential as a biomarker in NSCLC diagnosis and therapy.

## Data Availability Statement

All datasets generated for this study are included in the article/**Supplementary Material**.

## Ethics Statement

The studies involving human participants were reviewed and approved by the National Cancer Center/Cancer Hospital, Chinese Academy of Medical Sciences, and Peking Union Medical College. The patients/participants provided their written informed consent to participate in this study. The animal study was reviewed and approved by the National Cancer Center/Cancer Hospital, Chinese Academy of Medical Sciences, and Peking Union Medical College. Written informed consent was obtained from the individual(s) for the publication of any potentially identifiable images or data included in this article.

## Author Contributions

XL designed and performed experiments, analyzed data, and wrote the manuscript. FLv, FLi, MD, and YL participated in the experiments. SJ, ZL, and BZ collected clinical samples. BW provided a lot of animal experimental advice and guidance, and made detailed improvements to the manuscript. YG designed, conceived, and supervised the study and wrote the manuscript. All authors contributed to the article and approved the submitted version.

## Funding

The work was funded by the National key R&D Program of China (grant no. 2016YFC0901401).

## Conflict of Interest

The authors declare that the research was conducted in the absence of any commercial or financial relationships that could be construed as a potential conflict of interest.
