# Supplementary material for: LINC01089 Inhibits Tumorigenesis and Epithelial–Mesenchymal Transition of Non-small Cell Lung Cancer via the miR-27a/SFRP1/Wnt/β-catenin Axis
Source: Front Oncol. 2020 Nov 17;10:532581. doi: 10.3389/fonc.2020.532581 (PMC7705259; doi:10.3389/fonc.2020.532581)
Supplement: Supplementary file 1 [file DataSheet_1.docx]

**Figure S1. Validation of the gene expression and survival analysis of LINC01089 in NSCLC tissue samples and normal tissues from GEPIA.**

A. Box plots showing the LINC01089 expression level between LUAD samples and normal tissues from GEPIA. B. The expression of LINC01089 between LUSC samples and normal tissue using GEPIA. T, tumor tissues; N, normal tissues. Survival analysis of LINC01089 in NSCLC patients by GEPIA. C. The overall survival (OS) of LINC01089 in patients was analyzed by GEPIA. Kaplan–Meier curves showed the survival of NSCLC patients with the lower expressions of LINC01089 have a poor prognosis (log-rank test, *p < 0.01*). *Data are reported as means ± SD. *P<0.05.*

**Figure** **S2. Transfection efficiency of siLINC01089 and pcDNA3.1-LINC01089**

**(**A) RT-qPCR validated the expression level of LINC01089 knockdown by transfected with siLINC01089 in A549 cells. (B) RT-qPCR validated the expression level of LINC01089 by transfected with pcDNA3.1-LINC01089 in PC9 cells. (C) The data quantification of colony formation from Fig 3E. (D) The data quantification of colony formation from Fig 3F.

*Data are reported as means ± SD. **P<0.01; ***P<0.001.*

**Figure** **S3. CCk-8 assays and the transfection in NSCLC**

(A) CCK-8 assay of cell viability in A549 cells treated with siLINC01089 (B) CCK-8 assay of cell viability pcDNA3.1-LINC01089 in PC9 cells. (C) RT-qPCR validated the expression level of LINC01089 by transfected with lentivirus-mediated short hairpin (shRNA) or sh-NC in A549, H1299 and BEAS-2B cells. (D) CCK-8 assay of cell viability in BEAS-2B cells treated with shLINC01089 (E) RT-qPCR validated the expression level of LINC01089 by transfected with lentivirus-mediated pLV-LINC01089 or pLV-Empty in PC9 and H460 cells, H1299 and BEAS-2B cells. *Data are reported as means ± SD. **P<0.01; ***P<0.001.*

**Figure** **S4. Transfection efficiency of miR-27a inhibitor and mimics.**

RT-qPCR validated the expression level of miR-27a in A549 cells treated with miR-27a mimic or miR-27a inhibitor. *Data are mean ± SD of 3 independent experiments. ***P<0.001.*

**Figure** **S5. Western Blot assay of Wnt/β-catenin pathway in H1299 cells.**

Western blot was used to analyse the protein levels of p-GSK-3P and β-catenin in H1299 cells cotransfected with miR-27a inhibitor and/or siLINC01089. GAPDH acted as reference gene.
