# Supplementary figures and images for: LINC01089 Inhibits Tumorigenesis and Epithelial–Mesenchymal Transition of Non-small Cell Lung Cancer via the miR-27a/SFRP1/Wnt/β-catenin Axis
Source: Front Oncol. 2020 Nov 17;10:532581. doi: 10.3389/fonc.2020.532581 (PMC7705259; doi:10.3389/fonc.2020.532581)

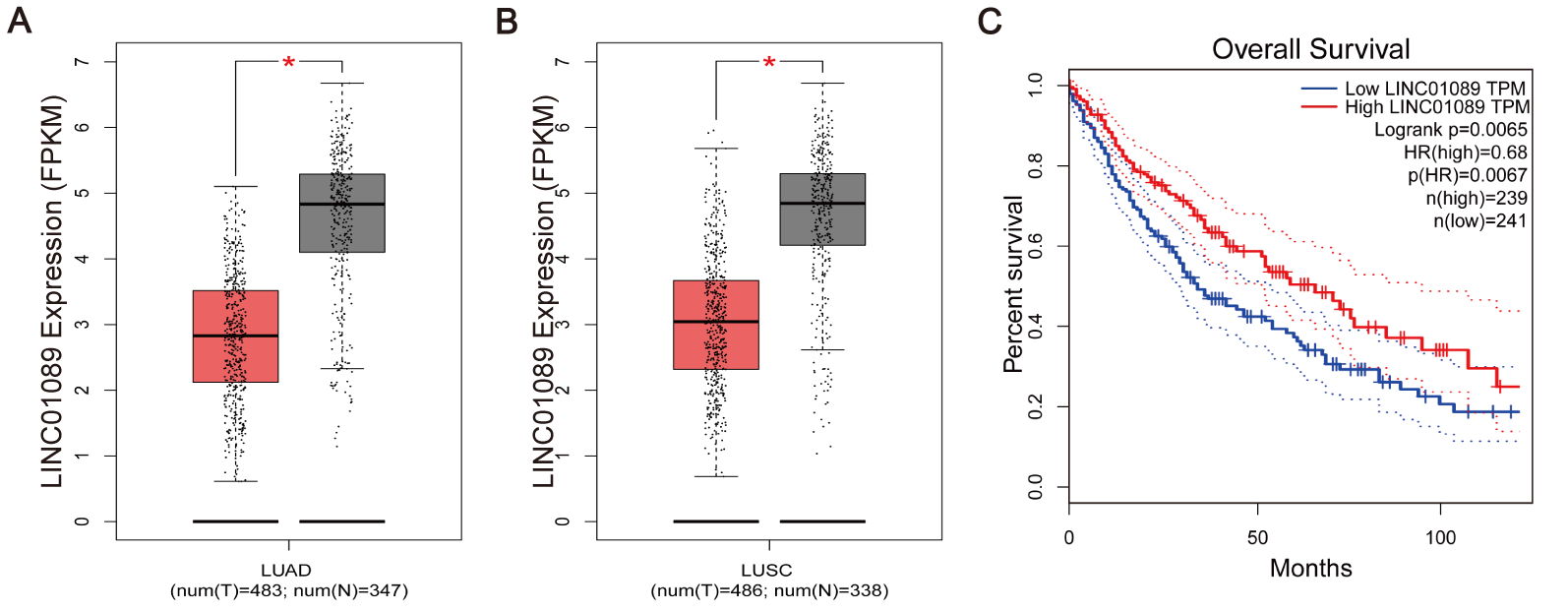

Supplement: Supplementary file 2 [file Image_1.tif]

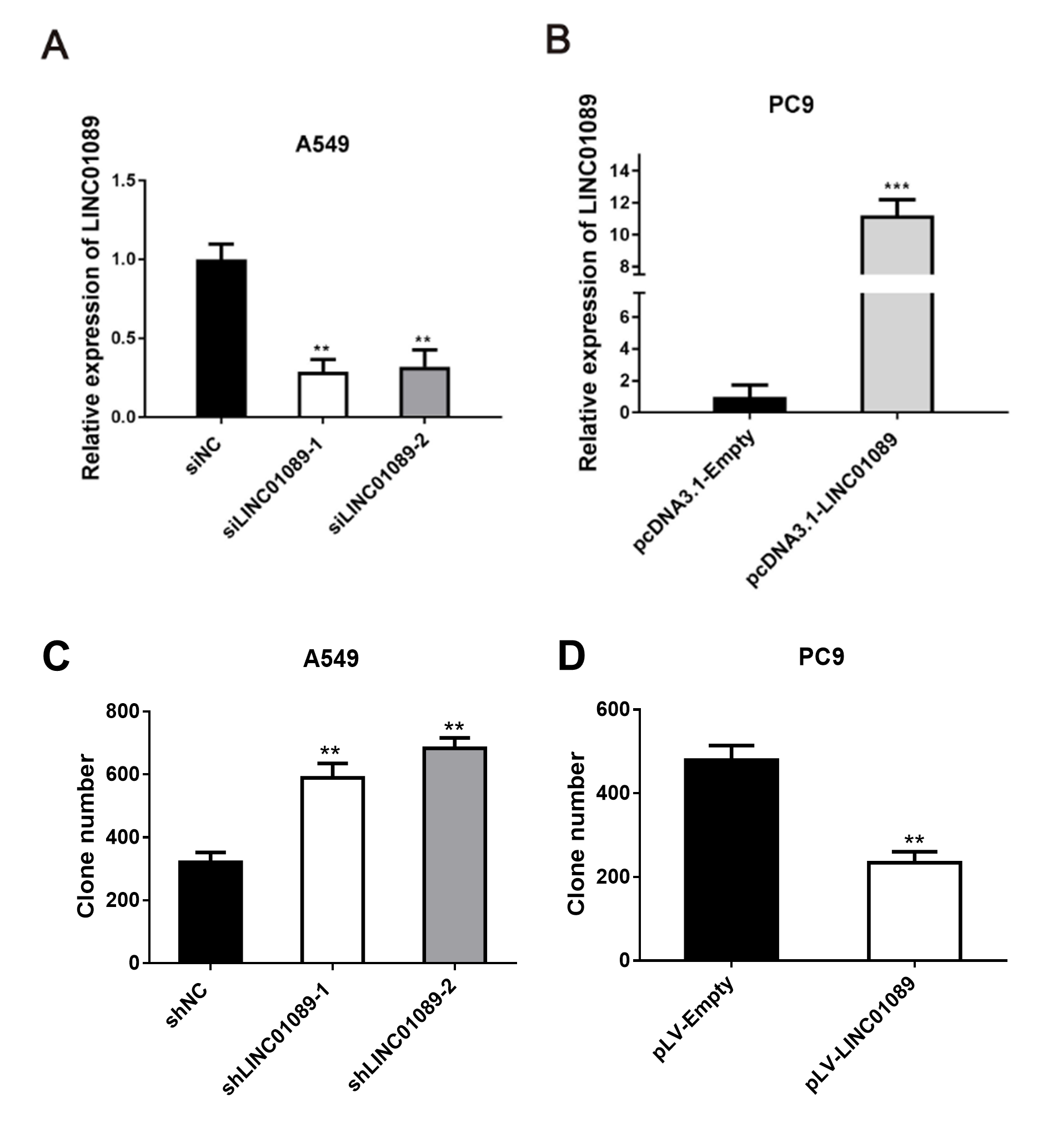

Supplement: Supplementary file 3 [file Image_2.tif]

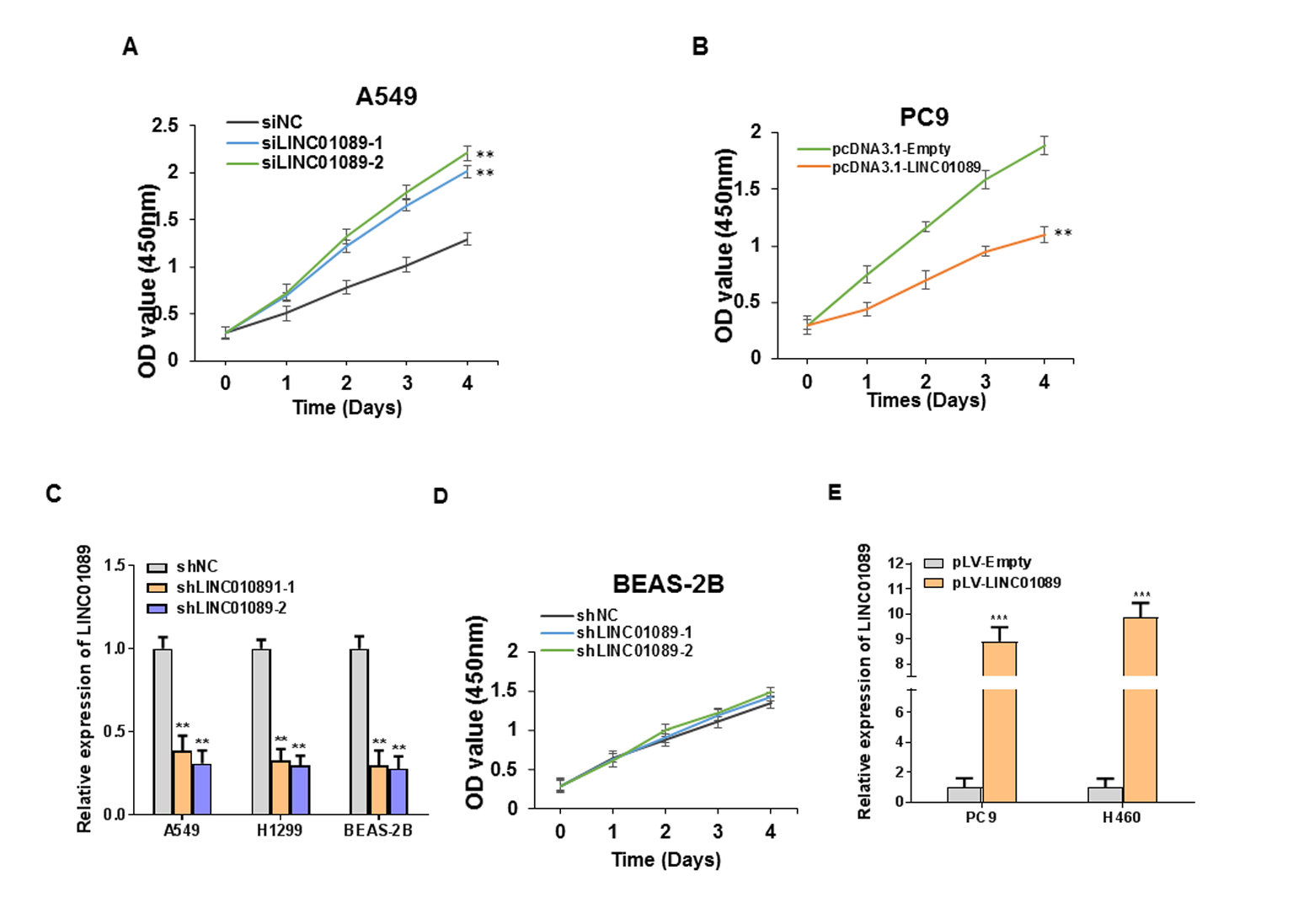

Supplement: Supplementary file 4 [file Image_3.tif]

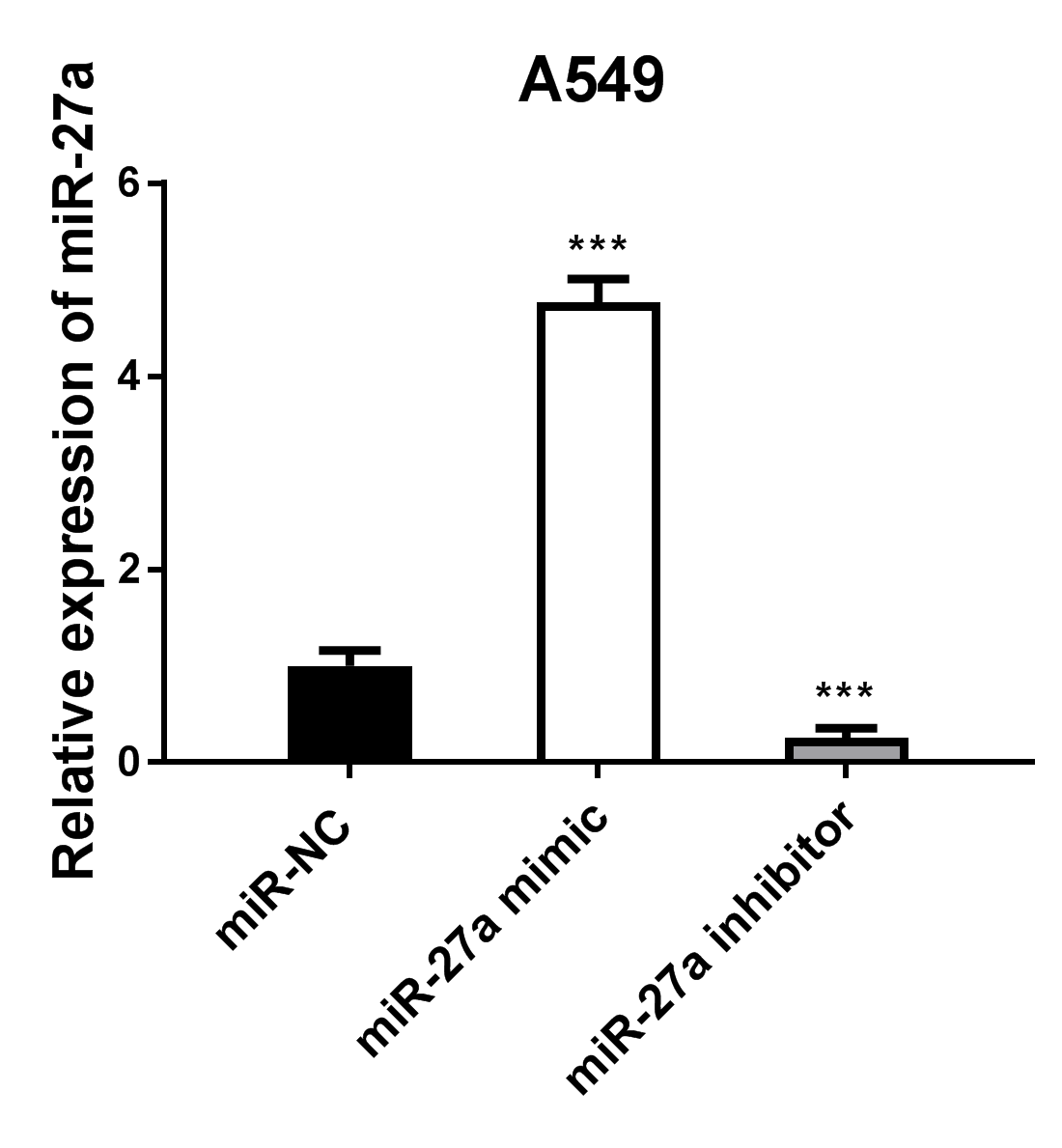

Supplement: Supplementary file 5 [file Image_4.tif]

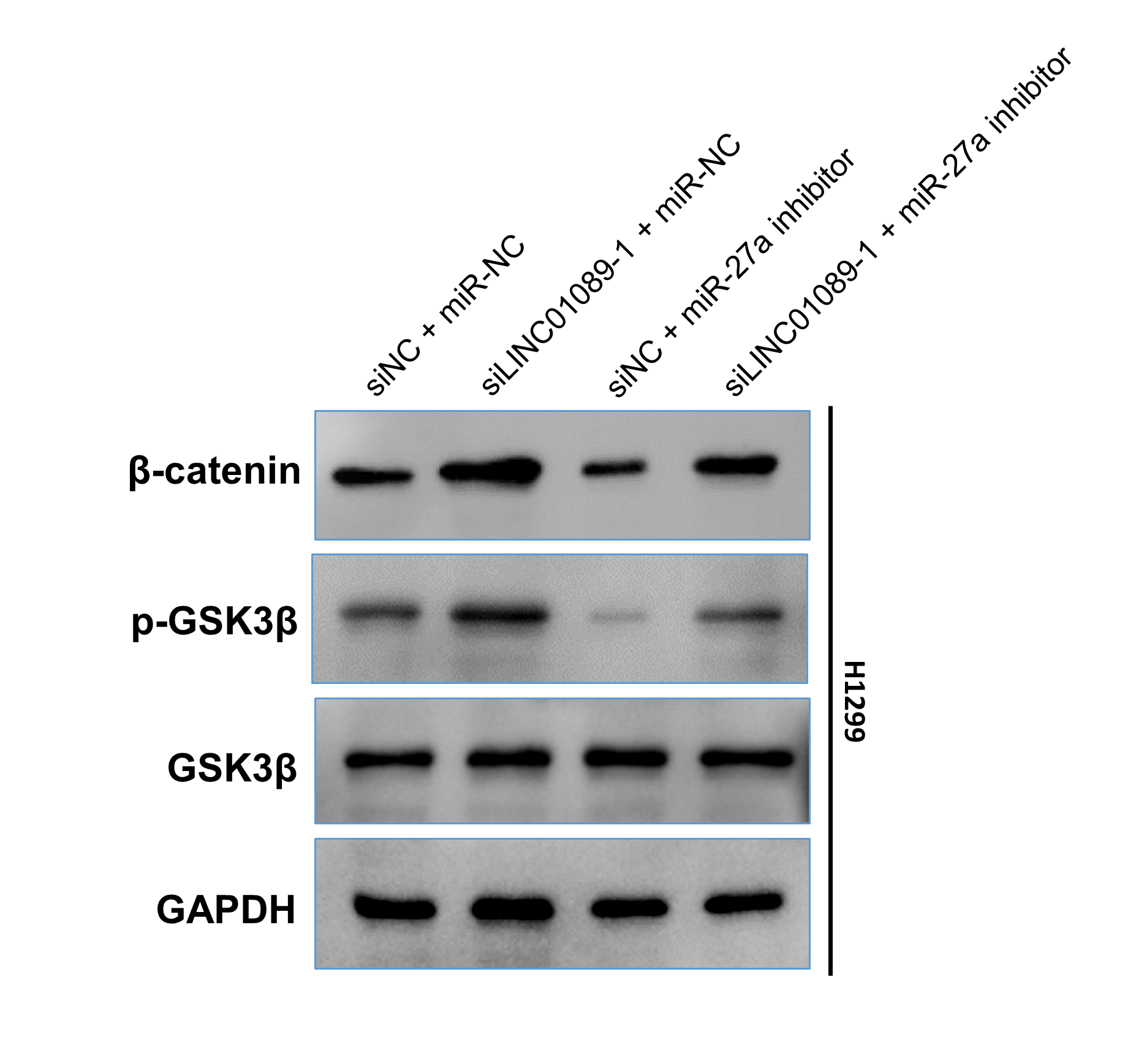

Supplement: Supplementary file 6 [file Image_5.tif]
